# Sulfotransferase SULT1A1 Arg213His Polymorphism with Cancer Risk: A Meta-Analysis of 53 Case-Control Studies

**DOI:** 10.1371/journal.pone.0106774

**Published:** 2014-09-16

**Authors:** Juanjuan Xiao, Yabiao Zheng, Yinghui Zhou, Ping Zhang, Jianguo Wang, Fangyuan Shen, Lixia Fan, Vijay Kumar Kolluri, Weiping Wang, Xiaolong Yan, Minghua Wang

**Affiliations:** 1 Department of Biochemical and Molecular Biology, Medical College, Soochow University, Suzhou, Jiangsu, China; 2 Department of Thoracic Surgery, Tangdu Hospital, Fourth Military Medical University, Xi'an, Shaanxi, China; Duke Cancer Institute, United States of America

## Abstract

**Background:**

The *SULT1A1* Arg213His (rs9282861) polymorphism is reported to be associated with many kinds of cancer risk. However, the findings are conflicting. For better understanding this SNP site and cancer risk, we summarized available data and performed this meta-analysis.

**Methods:**

Data were collected from the following electronic databases: PubMed, Web of Knowledge and CNKI. The association was assessed by odd ratio (OR) and the corresponding 95% confidence interval (95% CI).

**Results:**

A total of 53 studies including 16733 cancer patients and 23334 controls based on the search criteria were analyzed. Overall, we found *SULT1A1* Arg213His polymorphism can increase cancer risk under heterozygous (OR = 1.09, 95% CI = 1.01–1.18, P = 0.040), dominant (OR = 1.10, 95% CI = 1.01–1.19, P = 0.021) and allelic (OR = 1.08, 95% CI = 1.02–1.16, P = 0.015) models. In subgroup analyses, significant associations were observed in upper aero digestive tract (UADT) cancer (heterozygous model: OR = 1.62, 95% CI = 1.11–2.35, P = 0.012; dominant model: OR = 1.63, 95% CI = 1.13–2.35, P = 0.009; allelic model: OR = 1.52, 95% CI = 1.10–2.11, P = 0.012) and Indians (recessive model: OR = 1.93, 95% CI = 1.22–3.07, P = 0.005) subgroups. Hospital based study also showed marginally significant association. In the breast cancer subgroup, ethnicity and publication year revealed by meta-regression analysis and one study found by sensitivity analysis were the main sources of heterogeneity. The association between *SULT1A1* Arg213His and breast cancer risk was not significant. No publication bias was detected.

**Conclusions:**

The present meta-analysis suggests that *SULT1A1* Arg213His polymorphism plays an important role in carcinogenesis, which may be a genetic factor affecting individual susceptibility to UADT cancer. *SULT1A1* Arg213His didn't show any association with breast cancer, but the possible risk in Asian population needs further investigation.

## Introduction

Sulfotransferase (SULT) enzymes catalyze the sulfate conjugation of a broad range of substrates and play an important role in metabolism of endogenous and exogenous compounds including thyroid and steroid hormones, neurotransmitters, drugs and procarcinogens [1], [2]. There are many isoforms of the *SULT*s supergene family, each with different amino acid sequence identity and substrate specificity [3]. SULT1A1 is an important member of the sulfotransferase family involving in the pathogenic process of various cancers [3]–[5].

The *SULT1A1* gene is located on chromosome 16p12.1–p11.2 [6]. Previous study indicated that exon 7 of the *SULT1A1* gene contained a G to A transition at codon 213 (rs9282861) that causes an Arg to His amino acid substitution [4]. Some studies have shown that this genetic polymorphism leads to a decrease in enzymatic activity of SULT1A1 and the sulfonation efficiency thus associating with susceptibility to several cancers [7], [8]. Although the specific role of *SULT1A1* Arg213His polymorphism in carcinogenesis has been investigated in numerous case-control studies, the results have been inconclusive, even conflictive. In order to give a comprehensive and precise result, we performed this meta-analysis study to analyze the association between this polymorphism and cancer risk.

## Materials and Methods

### Identification of eligible studies

The meta-analysis was conducted following the criteria of Preferred Reporting Items for Systematic Reviews and Meta-Analyses (PRISMA) (Checklist S1). In this study, we did an exhaustive literature search on studies that examined the association of the *SULT1A1* gene polymorphisms with cancer risks. All eligible studies were identified by searching the following databases: PubMed, Web of Knowledge and China National Knowledge Infrastructure (CNKI, http://www.cnki.net/). The following terms were utilized: “sulfotransferase, *SULT* or *SULT1A1*”, “polymorphism, variation, variant or mutation” and “cancer or carcinoma”. In the CNKI database, we searched with these corresponding key words in Chinese characters. Included studies should meet the following criteria: (1) evaluating the association between *SULT1A1* Arg213His polymorphism and cancer risk; (2) study designed as case-control; (3) sufficient data available to estimate an odd ratio (OR) with its 95% confidence interval (95% CI).

### Data extraction

Two investigators extracted data independently and reached consensus on the following characteristics of the selected studies: first author's name, the year of publication, ethnicity of the study population, matching criteria, number of participants, genotype distribution and control source.

### Statistical analysis

Hardy-Weinberg equilibrium was assessed by Chi-square test. Crude odd ratio (OR) and 95% confidence interval (CI) were used to estimate the association between *SULT1A1* polymorphism and cancer susceptibility under the dominant model (Arg/His+His/His vs. Arg/Arg), recessive model (His/His vs. Arg/Arg_+_Arg/His), homozygous model (His/His vs. Arg/Arg), heterozygous model (His/Arg vs. Arg/Arg) and allelic model (His vs. Arg). The heterogeneity among the studies was evaluated by Q-test and *I^2^* value ranging from 0% to 100% to describe the percentage of between-study variation caused by heterogeneity. P value for the Q-test less than 0.10 indicates existing heterogeneity among studies. And then the pooled OR was measured by a random effect model (the DerSimonian-Laird method). Otherwise, a fixed effect model (the Mantel-Haenszel method) was chosen.

Subgroup analyses were performed according to cancer type (breast cancer, colorectal cancer, urothelial cancer, prostate cancer, lung cancer, upper aero digestive tract (UADT) cancer, ovarian cancer and gastric cancer), ethnicity (Caucasian, East Asian, Indian and African) and source of controls (hospital based and population based). When heterogeneity was detected, a multivariable meta-regression analysis including cancer type, ethnicity, control source and year of publication to explore potential source of heterogeneity and sensitivity analysis were performed.

The potential publication bias was estimated using Egger's linear regression test by visual inspection of the funnel plot. P_<_0.05 was considered statistically significant, and all P values were two-sided. Analyses were performed using the software Review Manager 5.3 (Cochrane Collaboration), R software (www.r-project.org) and STATA 12.0 software (StataCrop).

## Results

### Characteristics of eligible studies

**Figure 1:**
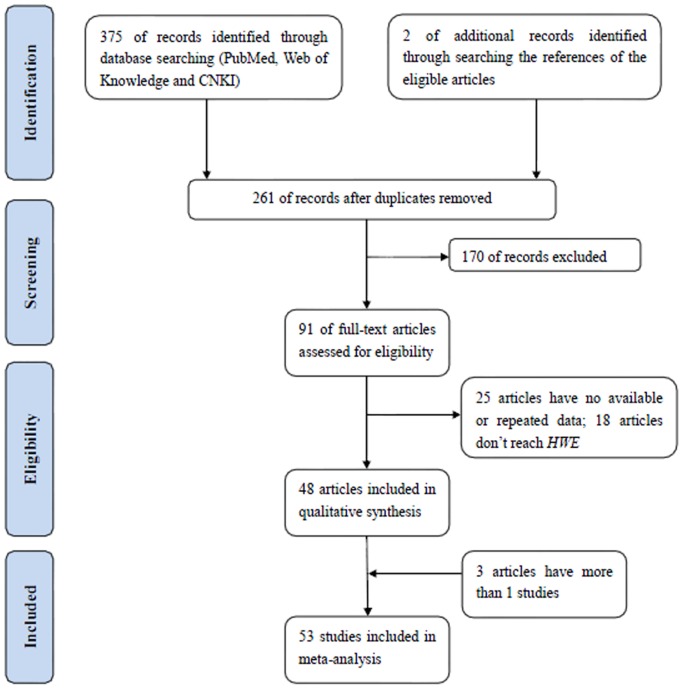
Flow diagram of the study selection process.

The flow diagram of literature search was given in Figure 1. A total of 91 studies focusing the association between the *SULT1A1* Arg213His polymorphism and cancer risks were identified. 25 of them were ruled out because of unavailable data or repeated data. Thus, the allele and genotype frequencies of the *SULT1A1* Arg213His polymorphism were extracted from 66 articles. However, 18 articles didn't meet with Hardy-Weinberg equilibrium and were abandoned (Excluded list S1). As a result, 53 studies of 48 articles, involving 16733 cases and 23334 controls were included in the pooled analyses [9]–[56].

The characteristics of studies included in the current meta-analysis are shown in Table 1. Among these studies, 13 were conducted for breast cancer, 10 for colorectal cancer, 7 for urothelial cancer, 5 for prostate cancer, 5 for lung cancer, 5 for UADT (upper aero digestive tract) cancer, 3 for ovarian cancer, 2 for gastric cancer and 1 for myeloid leukemia, multiple myeloma, and endometrial cancer, respectively. By ethnics, there were 27 studies of Caucasians, 11 studies of East Asians, 4 studies of Indians, 2 studies of Africans and 9 studies of mixed ethnics. By source of controls, 16 studies were population-based, 17 studies were hospital-based and 20 studies were not clear.

**Table 1:** Characteristics of studies included in the meta-analysis.

First author	Year	Cancer type	Ethnicity	Source of Control	Sample Size (Case/Control)	Genotype Distribution (Case/Control)			P for *HWE*
						Arg/Arg	Arg/His	His/His	
Seth	2000	Breast	Caucasian	Population	444/227	229/110	176/94	39/23	0.907
Steiner	2000	Prostate	Caucasian	Population	134/184	57/72	60/80	17/32	0.496
Bamber	2001	Colorectal	Caucasian	Mixed	226/293	96/137	104/124	26/32	0.885
Wang	2001	Lung	Caucasian	Population	463/485	195/226	201/196	67/63	0.148
Zheng	2001	Breast	Mixed	Population	155/328	55/148	71/136	29/44	0.368
Nowell	2002	Colorectal	Mixed	Population	130/301	48/101	67/145	15/55	0.973
Ozawa	2002	Urothelial	East Asians	Population	166/214	128/154	32/53	6/7	0.662
Sachse	2002	Colorectal	Caucasian	Population	490/593	217/275	209/255	64/63	0.944
Wong	2002	Colorectal	Caucasian	Unknown	383/402	175/178	179/190	29/34	0.239
Wu	2003	UADT	East Asians	Hospital	187/308	135/274	52/34	0/0	0.591
Tang	2003	Breast	Mixed	Unknown	103/133	50/79	42/47	11/7	1.000
Tsukino	2003	Urothelial	East Asians	Hospital	306/306	238/242	62/60	6/4	0.992
Zheng	2003	Urothelial	Mixed	Hospital	384/386	196/164	155/174	33/48	0.985
Chacko	2004	Breast	India	Hospital	140/140	76/95	56/41	8/4	0.986
Hung	2004	Urothelial	Caucasian	Hospital	201/214	121/116	72/88	8/10	0.422
Langsenlehner	2004	Breast	Caucasian	Population	498/499	201/224	250/212	47/63	0.515
Liang	2004	Lung	East Asians	Population	805/809	581/672	217/134	7/3	0.397
Nowell	2004	Prostate	African	Population	106/93	59/46	42/41	5/6	0.732
Nowell	2004	Prostate	Caucasian	Population	344/310	149/109	149/145	46/56	0.815
Cheng	2005	Breast	East Asians	Hospital	468/740	439/693	27/47	2/0	0.672
Jerevall	2005	Breast	Caucasian	Population	229/227	80/83	121/106	28/38	0.916
Lilla	2005	Breast	Caucasian	Population	419/884	198/374	169/403	52/107	0.995
Pereira	2005	Colorectal	Mixed	Unknown	42/100	15/45	23/44	4/11	0.999
Pereira	2005	Gastric	Mixed	Unknown	20/100	10/45	8/44	2/11	0.999
Pereira	2005	Myeloid leukemia	Mixed	Unknown	35/100	14/45	16/44	5/11	0.999
Pereira	2005	Multiple myeloma	Mixed	Unknown	28/100	7/45	15/44	6/11	0.999
Sellers	2005	Ovary	Caucasian	Hospital	454/542	197/236	194/237	63/69	0.735
Sillanpaa	2005	Breast	Caucasian	Population	480/478	145/147	229/221	106/110	0.313
Sun	2005	Colorectal	Caucasian	Population	109/666	43/266	27/303	39/97	0.778
Boccia	2006	UADT	Caucasian	Hospital	123/247	71/156	44/82	8/9	0.907
Chen	2006	Colorectal	East Asians	Population	83/343	67/301	15/41	1/1	0.950
Feng	2006	UADT	East Asians	Hospital	163/166	109/129	50/32	4/5	0.258
Boccia	2007	Gastric	Caucasian	Hospital	107/254	57/156	39/85	11/13	0.950
Holt	2007	Ovary	African	Population	33/127	21/67	10/48	2/12	0.735
Holt	2007	Ovary	Caucasian	Population	277/448	117/185	133/213	27/50	0.624
Lilla	2007	Colorectal	Caucasian	Population	504/603	212/263	225/259	67/81	0.404
Roupret	2007	Urothelial	Caucasian	Hospital	268/268	119/140	99/101	50/27	0.395
Hirata	2008	Endometrial	Caucasian	Hospital	150/165	68/103	59/52	23/10	0.619
Koike	2008	Prostate	East Asians	Hospital	126/119	94/85	32/32	0/2	0.875
Wang	2008	Urothelial	East Asians	Hospital	300/300	261/240	37/54	2/6	0.377
Arslan	2009	Lung	Caucasian	Population	106/271	50/162	52/99	4/10	0.554
Cleary	2010	Colorectal	Caucasian	Population	1164/1292	544/598	502/540	118/154	0.173
MERIE-GENICA	2010	Breast	Caucasian	Population	3139/5426	1381/2338	1332/2430	426/658	0.789
Syamala	2010	Breast	India	Population	359/367	254/271	87/90	18/6	0.894
Arslan	2011	Prostate	Caucasian	Population	104/151	55/91	38/54	11/6	0.846
Ihsan	2011	Lung	India	Population	188/290	123/153	50/116	15/21	0.988
Serrano	2011	Breast	Caucasian	Hospital	46/136	24/71	18/55	4/10	0.989
Tamaki	2011	Lung	East Asians	Hospital	192/203	120/132	70/68	2/3	0.211
Cui	2012	Urothelial	East Asians	Hospital	282/257	218/201	59/52	5/4	0.956
Eichholzer	2012	Colorectal	Caucasian	Population	424/819	183/389	193/354	48/76	0.940
Khvostova	2012	Breast	Caucasian	Population	335/530	47/166	164/261	124/103	1.000
Kotnis	2012	UADT	India	Unknown	109/194	60/132	43/60	6/2	0.232
Santos	2012	UADT	Mixed	Hospital	202/196	94/94	89/82	19/20	0.944

*HWE*, Hardy-Weinberg equilibrium.

### Overall Analysis

Table 2 showed the results of overall analysis and the subgroup analysis. The analyses on the full data set indicated a significant association of the *SULT1A1* Arg213His polymorphism with cancer risk: heterozygous (OR = 1.09, 95% CI = 1.01–1.19, P = 0.035), homozygous (OR = 1.20, 95% CI = 1.04–1.39, P = 0.014), dominant (OR = 1.12, 95% CI = 1.03–1.22, P  =  0.008) (Figure S1), recessive (OR = 1.16, 95% CI = 1.02–1.32, P = 0.027) and allelic model (OR = 1.11, 95% CI = 1.04–1.20, P = 0.003), with high heterogeneity among studies (*I^2^* = 63.1%, 62.6%, 68.5%, 58.3% and 73.7%, respectively, all P<0.001)(Table 3).

**Table 2:** Overall and subgroup meta-analysis of the association between *SULT1A1* Arg213His polymorphism and cancer risk under genetic models.

Groups	N	Cases/Controls	Heterozygous		Homozygous		Dominant		Recessive		Allelic	
			OR (95% CI)	P	OR (95% CI)	P	OR (95% CI)	P	OR (95% CI)	P	OR (95% CI)	P
**Total**	53	16733/23334	1.09 [1.01, 1.19]a	0.035	1.20 [1.04, 1.39]a	0.014	1.12 [1.03, 1.22]a	0.008	1.16 [1.02, 1.32]a	0.027	1.11 [1.04, 1.20]a	0.003
**Cancer type**												
Breast cancer	13	6815/10115	1.14 [0.97, 1.33]a	0.108	1.37 [1.01, 1.87]a	0.045	1.18 [1.00, 1.40]a	0.050	1.23 [0.96, 1.57]a	0.108	1.15 [1.00, 1.32]a	0.044
Colorectal cancer	10	3555/5412	1.05 [0.95, 1.15]b	0.354	1.13 [0.88, 1.45]a	0.352	1.06 [0.97, 1.15]b	0.224	1.13 [0.83, 1.52]a	0.439	1.08 [0.97, 1.19]a	0.169
Urothelial cancer	7	1907/1945	0.86 [0.74, 1.00]b	0.050	0.97 [0.56, 1.71]a	0.925	0.88 [0.71, 1.10]a	0.269	1.03 [0.63, 1.69]a	0.907	0.92 [0.73, 1.16]a	0.475
Prostate cancer	5	814/857	0.87 [0.70, 1.07]b	0.188	0.82 [0.44, 1.51]a	0.515	0.85 [0.69, 1.03]b	0.097	0.79 [0.58, 1.08]b	0.145	0.86 [0.74, 1.00]b	0.051
Lung cancer	5	1754/2058	1.19 [0.79, 1.80]a	0.404	1.19 [0.87, 1.63]b	0.269	1.21 [0.82, 1.79]a	0.344	1.15 [0.85, 1.56]b	0.357	1.18 [0.88, 1.58]a	0.279
UADT cancer	5	784/1111	1.62 [1.11, 2.35]a	0.012	1.39 [0.85, 2.26]b	0.185	1.63 [1.13, 2.35]a	0.009	1.28 [0.80, 2.05]b	0.307	1.52 [1.10, 2.11]a	0.012
Ovarian cancer	3	764/1117	0.96 [0.79, 1.17]b	0.697	0.97 [0.72, 1.32]b	0.857	0.96 [0.80, 1.16]b	0.695	0.99 [0.74, 1.32]b	0.944	0.98 [0.85, 1.12]b	0.746
Gastric cancer	2	127/354	1.16 [0.75, 1.80]b	0.510	1.81 [0.86, 3.81]b	0.12	1.26 [0.84, 1.91]b	0.264	1.73 [0.84, 3.57]b	0.139	1.29 [0.93, 1.78]b	0.126
**Ethnicity**												
Caucasian	27	11621/16614	1.06 [0.97, 1.16]a	0.174	1.20 [1.01, 1.43]a	0.035	1.10 [1.00, 1.20]a	0.044	1.16 [0.99, 1.36]a	0.058	1.10 [1.01, 1.19]a	0.019
East Asian	11	3078/3765	1.22 [0.92, 1.61]a	0.175	1.12 [0.71, 1.79]b	0.626	1.21 [0.92, 1.61]a	0.176	1.10 [0.69, 1.75]b	0.697	1.18 [0.92, 1.52]a	0.187
Indian	4	796/991	1.09 [0.66, 1.80]a	0.748	2.25 [0.94, 5.37]a	0.067	1.19 [0.72, 1.96]a	0.500	1.93 [1.22, 3.07]b	0.005	1.25 [0.84, 1.85]a	0.274
African	2	139/220	0.75 [0.47, 1.21]b	0.239	0.60 [0.23, 1.58]b	0.299	0.73 [0.46, 1.15]b	0.173	0.68 [0.26, 1.75]b	0.420	0.77 [0.53, 1.11]b	0.158
**Source of controls**												
Hospital based	17	3895/4718	1.17 [1.00, 1.38]a	0.056	1.38 [1.12, 1.68]b	0.002	1.21 [1.02, 1.43]a	0.029	1.31 [1.08, 1.59]b	0.006	1.19 [1.03, 1.38]a	0.020
Population based	16	8295/12176	0.94 [0.85, 1.03]a	0.200	0.98 [0.83, 1.17]a	0.855	0.96 [0.91, 1.02]b	0.162	1.02 [0.85, 1.24]a	0.825	0.98 [0.90, 1.06]a	0.584

N: total number of studies involved in the analysis; a: random effect model; b: fix effect model.

**Table 3:** The overall and subgroup heterogeneity test of the *SULT1A1* Arg213His polymorphism on cancer risk.

Groups	Heterozygous		Homozygous		Dominant		Recessive		Allelic	
	*I^2^* (%)	P	*I^2^* (%)	P	*I^2^* (%)	P	*I^2^* (%)	P	*I^2^* (%)	P
**Total**	63.1	0.000	62.6	0.000	68.5	0.000	58.3	0.000	73.7	0.000
**Cancer type**										
Breast cancer	67.1	0.000	79.4	0.000	75.9	0.000	72.7	0.000	80.7	0.000
Colorectal cancer	16.4	0.354	58.2	0.010	0.00	0.659	74.0	0.000	50.8	0.032
Urothelial cancer	20.6	0.272	62.7	0.013	57.9	0.027	54.0	0.042	73.0	0.001
Prostate cancer	0.00	0.719	53.8	0.070	12.9	0.332	45.8	0.117	47.5	0.107
Lung cancer	86.2	0.000	0.00	0.665	85.8	0.000	0.00	0.841	83.0	0.000
UADT cancer	68.5	0.013	44.9	0.142	69.1	0.012	42.0	0.160	72.1	0.006
Ovarian cancer	0.00	0.673	0.00	0.562	0.00	0.560	0.00	0.603	0.00	0.460
Gastric cancer	0.00	0.457	16.8	0.273	0.00	0.325	0.00	0.347	27.9	0.239
**Ethnicity**										
Caucasian	51.5	0.001	72.7	0.000	62.5	0.000	70.9	0.000	74.4	0.000
East Asian	77.8	0.000	0.00	0.481	78.8	0.000	0.00	0.547	77.8	0.000
Indian	81.9	0.001	62.7	0.045	83.0	0.001	42.6	0.156	81.0	0.001
African	0.00	0.724	0.00	0.845	0.00	0.685	0.00	0.882	0.00	0.653
**Source of controls**										
Hospital based	58.6	0.001	32.4	0.103	64.6	0.000	21.7	0.207	68.0	0.000
Population based	40.7	0.046	57.4	0.002	31.1	0.114	69.1	0.000	55.7	0.004

### Subgroup Analyses

**Figure 2:**
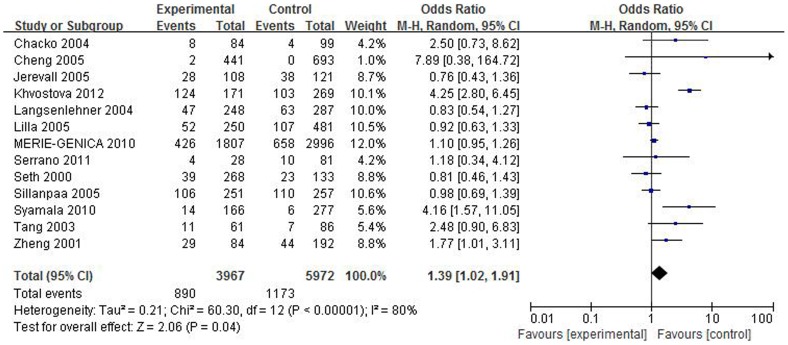
Forest plot on the association between *SULT1A1* Arg213His polymorphism and breast cancer risk in homozygous model.

**Figure 3:**
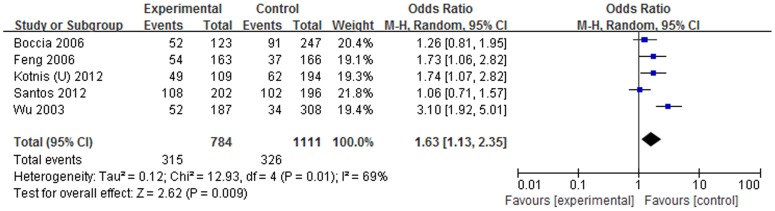
Forest plot on the association between *SULT1A1* Arg213His polymorphism and UADT cancer risk in dominant model.

We analyzed the association in cancer type subgroup. *SULT1A1* Arg213His polymorphism can increase cancer risks in the following cancer types: breast cancer (homozygous model: OR = 1.37, 95% CI = 1.01–1.87, P = 0.045; dominant model: OR = 1.18, 95% CI = 1.00–1.40, P = 0.050 and allelic model: OR = 1.15, 95% CI = 1.00–1.32, P = 0.044); UADT cancer (heterozygous model: OR = 1.62, 95% CI = 1.11–2.35, P = 0.012; dominant model: OR = 1.63, 95% CI = 1.13–2.35, P = 0.009 and allelic model: OR = 1.52, 95% CI = 1.10–2.11, P = 0.012). Forest plots of breast cancer risk and UADT cancer risk were shown in Figure 2 and Figure 3 separately.

Analyzed by ethnicity, a moderately increased risk was observed in Caucasians (homozygous model: OR = 1.20, 95% CI = 1.01–1.43, P = 0.035 and allelic model: OR = 1.10, 95% CI = 1.01–1.19, P = 0.019) and Indians (recessive model: OR = 1.93, 95% CI = 1.22–3.07, P = 0.005). No significant association was found in other ethnicities in any model.

By control source, significant association was observed in hospital based study, but not the population based study.

### Meta-regression analysis

To find potential source of heterogeneity, multivariable meta-regression analyses were conducted in total group and subgroups including cancer type, ethnicity, control source and publication year. In the breast cancer subgroup, ethnicity (heterozygous model, P = 0.027; recessive model, P = 0.020) and publication year (heterozygous model, P = 0.019; recessive model, P = 0.012) are significant sources of heterogeneity (Table S1). Other variables don't affect heterogeneity.

### Sensitivity analysis

**Figure 4:**
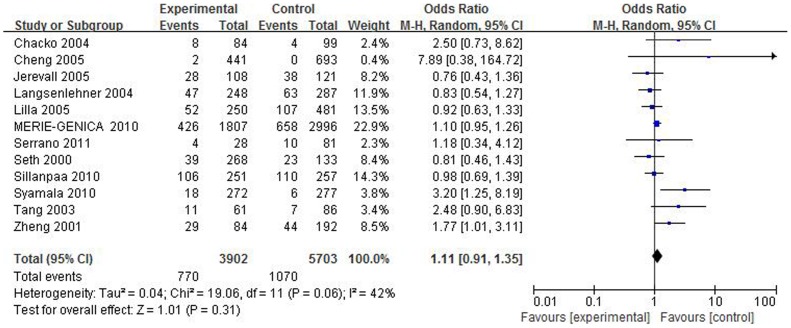
Forest plot on the association between *SULT1A1* Arg213His polymorphism and breast cancer risk in homozygous model omitting Khvostova's study.

The sensitivity analysis was constructed by repeating the meta-analysis sequentially removing each study. In the recessive model, two studies [26], [57] were found to affect the pooled OR and the heterogeneity when removed. The study conducted by Khvostova was focused on breast cancer and Sun's study was focused on colorectal cancer among Caucasians, so further sensitivity analyses were conducted in total data set and breast cancer, colorectal cancer and Caucasian subgroups after removing the two studies (Table 4 and Table S2). In total group, the heterogeneity was significantly decreased (*I^2^* = 58.2, 42.2, 63.5, 33.1 and 66.4, respectively). In the subgroup sensitivity analyses, removing the two studies can significantly decrease the heterogeneity among studies, most *I^2^* values less than 50%. And this polymorphism didn't show any obvious correlation with breast cancer risk (Figure 4). At last, we conducted the sensitivity analyses on the remaining studies and the result was stable.

**Table 4:** Meta-analysis in breast, colorectal and Caucasian subgroups after omitting studies of Khvostova and Sun.

Groups	Heterozygous		Homozygous		Dominant		Recessive		Allelic	
	OR (95% CI)	P	OR (95% CI)	P	OR (95% CI)	P	OR (95% CI)	P	OR (95% CI)	P
**Total**	1.09 [1.01, 1.18]a	0.040	1.10 [0.97, 1.24]a	0.131	1.10 [1.01, 1.19]a	0.021	1.06 [0.96, 1.18]a	0.261	1.08 [1.02, 1.16]a	0.015
**Cancer type**										
Breast cancer	1.05 [0.93, 1.19]a	0.400	1.11 [0.91, 1.35]a	0.312	1.07 [0.95, 1.20]a	0.256	1.07 [0.89, 1.30]a	0.469	1.06 [0.97, 1.15]a	0.219
Colorectal cancer	1.07 [0.97, 1.18]b	0.165	1.00 [0.86, 1.16]b	0.997	1.06 [0.97, 1.16]b	0.226	0.97 [0.84, 1.12]b	0.439	1.02 [0.96, 1.10]b	0.439
**Ethnicity**										
Caucasian	1.01 [0.96, 1.07]b	0.690	1.07 [0.94, 1.21]a	0.308	1.05 [0.98, 1.13]a	0.169	1.04 [0.93, 1.17]a	0.470	1.05 [0.98, 1.11]a	0.160

### Publication bias

**Figure 5:**
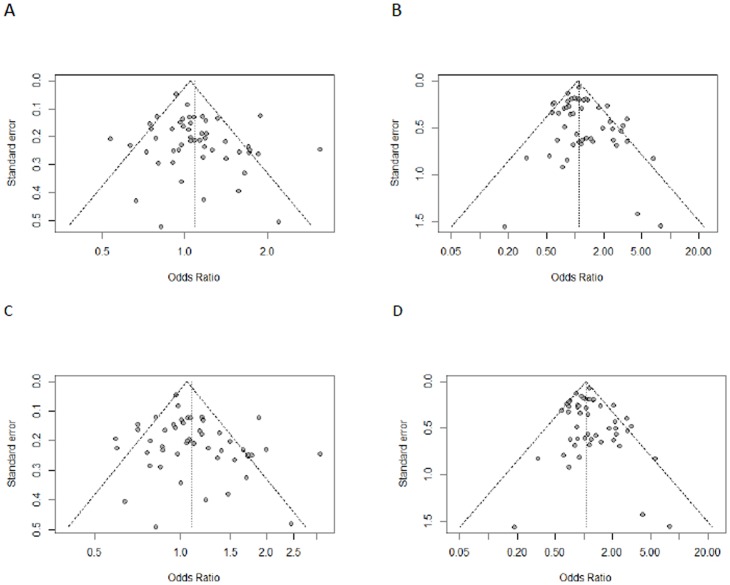
Begg's funnel plot of the Egger's test for publication bias of SULT1A1 Arg213His polymorphism and cancer risk. (A) heterozygous model (B) homozygous model (C) dominant model (D) recessive model The horizontal line in the funnel plot indicates the fixed-effects summary estimate, whereas the sloping lines indicate the expected 95% confidence intervals for a given SE.

Funnel plots and Egger's test were carried out to assess publication bias. The shapes of funnel plots indicated no obvious asymmetry (Figure 5). Egger's test found no publication bias in the heterozygous (P = 0.074); homozygous (P = 0.146); dominant (P = 0.076); recessive (P = 0.282) and allelic model (P = 0.081).

## Discussion

SULT1A1 enzyme encoded by *SULT1A1* gene plays an important role in xenobiotic metabolism. The Arg213His polymorphism, the most widely studied polymorphism within *SULT1A1* gene, can reduce enzyme activity and thermostability, and consequently results in an individual's susceptibility to cancer [7], [8].

There have been a few meta-analyses focusing on this mutation and cancer risk [58]–[60]. However, most of these analyses were conducted before the year 2012 and a new meta-analysis is needed to give a comprehensive conclusion due to the increasing data of case-control studies.

This present meta-analysis, including 16733 cases and 23334 controls from 53 case-control studies, explored the association between the *SULT1A1* Arg213His polymorphism and cancer risk. This is the largest scale meta-analysis so far. Our results suggested that the *SULT1A1* Arg213His was associated with UADT cancer risk. As the upper aero digestive tract is exposed to numerous potential carcinogens such as phenolic xenobiotics, polycyclic aromatic hydrocarbons and heterocyclic aromatic amines contained in cigarette smoking, environmental pollutants and some food, this result manifests that the mutation within *SULT1A1* causes the low SULT1A1 activity and is associated with high susceptibility to cancers related with environment.

In the sensitivity analyses, the study conducted by Khvostova influences the pooled estimates and the heterogeneity most in breast cancer subgroup. And after removing this study, the significant association between *SULT1A1* Arg213His and breast cancer risk became null (Figure 2 and Figure 4). We further checked data from Khvostova and observed the percentage of wild homozygous genotype in Khvostova's study was obviously lower than that in other studies thus causing great heterogeneity. At last a robust result was achieved and failed to reveal significant association in breast cancer subgroup. This result is similar to Wang, Lee and Jiang [61]–[63], but they found a positive association of this polymorphism with breast cancer susceptibility among Asians. While in our meta-analysis, we only recruited one paper focused on breast cancer among Asians because other papers on Asians deviate from *HWE* and were excluded. This is a limitation of this meta-analysis and more independent case-control studies conducted on Asians are needed to conclude a more comprehensive result.

In the ethnic subgroup analysis, we found that the genotype distributions of the SNP site are different in ethnic groups. When calculating the percentage of alleles in every ethnic, we found that His allele in Asians (9.58%) is significantly less than in Caucasians (35.2%). Different ethnicities may have different genetic backgrounds, thus causing different genotype frequencies in Asian and other ethnic groups which may influence cancer susceptibility.

Li and Kotnis have conducted meta-analyses focused on environment-related cancers, such as tobacco-related cancers and found cancer risk could be modulated by interaction between genetic variants and environmental factors [58], [59]. As exposed environmental factors are different according to cancer types, for example smoking leads to lung cancer, while the intake of meat influences breast cancer and colorectal cancer [64], [65] and our analysis took many kinds of cancer into account, we decided not to include environmental factors. Moreover, the definitions of exposed environmental factors were not consistent in the studies, which could cause great heterogeneity. Our estimates were based on crude OR values, not adjusted OR values, which may yield inaccurate calculation.

There were several sources bringing in heterogeneity, such as study design, age and sex distribution, and ethnicity. Meta-regression analysis was conducted to find source of heterogeneity. In the breast cancer subgroup, publication year could cause great heterogeneity and further attention was paid to years. We found all the recruited studies were carried out before 2005 or after 2010, and there were no studies between 2006 and 2009. The His allele was 29.6% in the studies before 2005 and 33.0% after 2010, which was significantly different (P = 0.02). This may be caused by the different study population, and needs more case-control studies to illustrate.

In conclusion, our meta-analysis suggests that the *SULT1A1* Arg213His polymorphism may contribute UADT cancer risk. As the result was calculated through sampling statics and statistical difference is not the same as clinical difference, the result can be used for clinical reference, not for clinical diagnosis of cancer. Further detailed investigation with larger number of worldwide participants is needed to clarify the role of this polymorphism in cancer risk.

## Supporting Information

Figure S1 Forest plot on the association between *SULT1A1* Arg213His polymorphism and overall cancer risk in dominant model.
(TIF)

Table S1 The P-value of meta-regression in overall and breast cancer groups.
(DOCX)

Table S2 Heterogeneity test after omitting studies of Khvostova and Sun.
(DOCX)

Checklist S1 PRISMA 2009 Checklist.
(DOC)

Excluded list S1 Excluded studies list with reasons.
(XLS)
