# Supplementary figures and images for: Sulfotransferase SULT1A1 Arg213His Polymorphism with Cancer Risk: A Meta-Analysis of 53 Case-Control Studies
Source: PLoS One. 2014 Sep 16;9(9):e106774. doi: 10.1371/journal.pone.0106774 (PMC4165769; doi:10.1371/journal.pone.0106774)

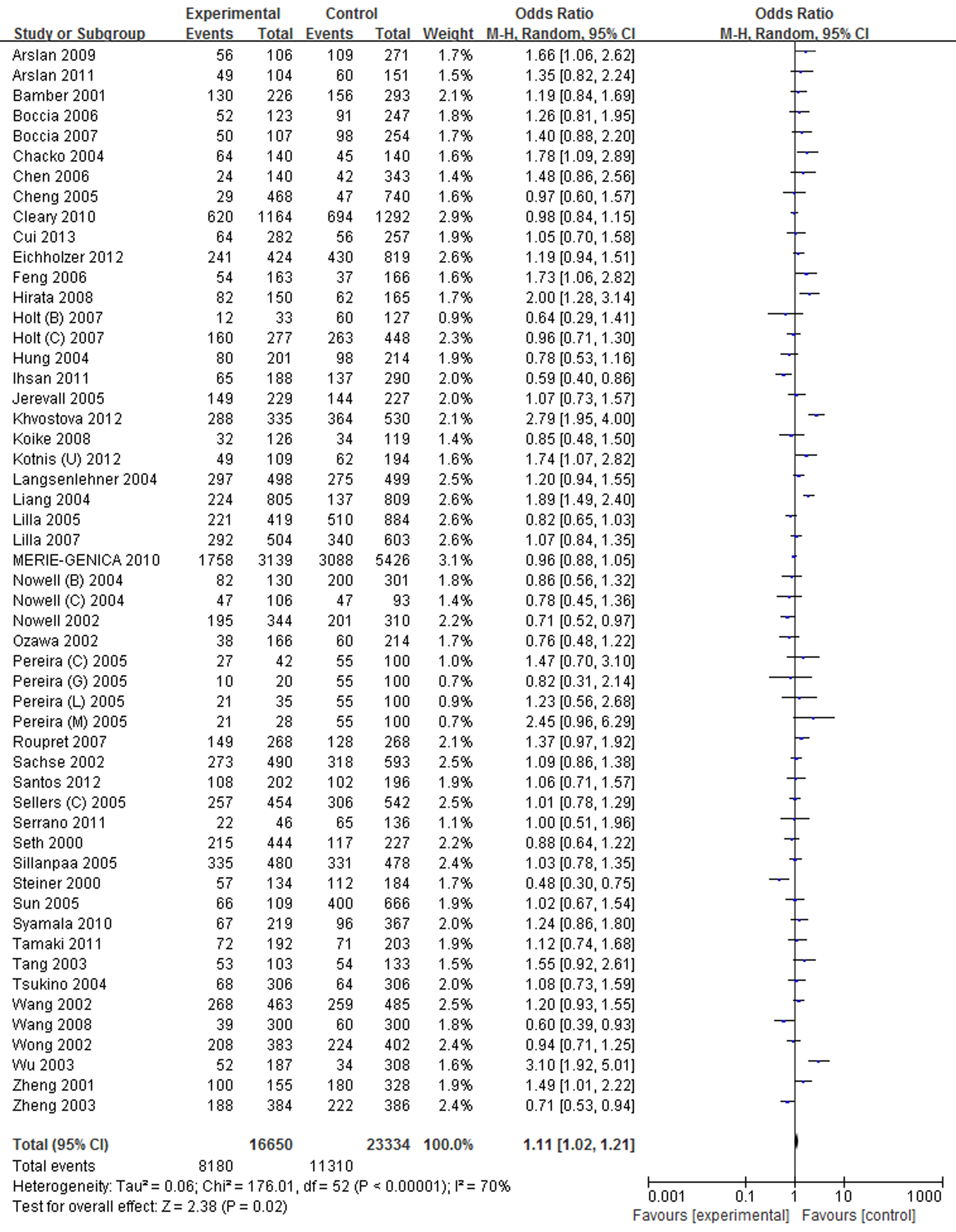

Supplement: Figure S1 — Forest plot on the association between SULT1A1 Arg213His polymorphism and overall cancer risk in dominant model. (TIF) [file pone.0106774.s001.tif]
