# Supplementary material for: Sulfotransferase SULT1A1 Arg213His Polymorphism with Cancer Risk: A Meta-Analysis of 53 Case-Control Studies
Source: PLoS One. 2014 Sep 16;9(9):e106774. doi: 10.1371/journal.pone.0106774 (PMC4165769; doi:10.1371/journal.pone.0106774)
Supplement: Table S1 — The P-value of meta-regression in overall and breast cancer groups. (DOCX) [file pone.0106774.s002.docx]

Table S1. The P-value of meta-regression in overall and breast cancer groups

| **Groups** | **Variables** | **Heterozygous** | **Homozygous** | **Dominant** | **Recessive** |
| --- | --- | --- | --- | --- | --- |
| Total | Publication year | 0.925 | 0.055 | 0.600 | 0.071 |
|  | Cancer type | 0.197 | 0.551 | 0.231 | 0.555 |
|  | Ethnicity | 0.870 | 0.763 | 0.728 | 0.724 |
|  | Control source | 0.514 | 0.602 | 0.514 | 0.846 |
| Breast cancer | Publication year | 0.528 | 0.019 | 0.236 | 0.012 |
|  | Ethnicity | 0.328 | 0.027 | 0.175 | 0.020 |
|  | Control source | 0.240 | 0.875 | 0.320 | 0.817 |
