# Supplementary material for: Sulfotransferase SULT1A1 Arg213His Polymorphism with Cancer Risk: A Meta-Analysis of 53 Case-Control Studies
Source: PLoS One. 2014 Sep 16;9(9):e106774. doi: 10.1371/journal.pone.0106774 (PMC4165769; doi:10.1371/journal.pone.0106774)
Supplement: Table S2 — Heterogeneity test after omitting studies of Khvostova and Sun. (DOCX) [file pone.0106774.s003.docx]

Table S2. Heterogeneity test after omitting studies of Khvostova and Sun

| Groups | Heterozygous | | Homozygous | | Dominant | | Recessive | | Allelic | |
| --- | --- | --- | --- | --- | --- | --- | --- | --- | --- | --- |
|  | *I^2^* (%) | P | *I^2^* (%) | P | *I^2^* (%) | P | *I^2^* (%) | P | *I^2^* (%) | P |
| **Total** | 58.2 | 0.000 | 42.2 | 0.001 | 63.5 | 0.000 | 33.1 | 0.014 | 66.4 | 0.000 |
| **Cancer type** | |  |  |  |  |  |  |  |  |  |
| Breast cancer | 43.5 | 0.053 | 42.3 | 0.060 | 42.8 | 0.057 | 44.9 | 0.046 | 41.9 | 0.062 |
| Colorectal cancer | 0.00 | 0.815 | 16.7 | 0.294 | 0.00 | 0.562 | 10.9 | 0.344 | 24.0 | 0.230 |
| **Ethnicity** |  |  |  |  |  |  |  |  |  |  |
| Caucasian | 24.2 | 0.136 | 44.9 | 0.009 | 39.6 | 0.023 | 40.7 | 0.019 | 52.0 | 0.001 |
